# An evaluation of the effectiveness of platelet rich plasma epidural injections for low back pain suspected to be of disc origin – A pilot study with one-year follow-up

**DOI:** 10.1016/j.inpm.2024.100403

**Published:** 2024-04-03

**Authors:** David Playfair, Ashley Smith, Robert Burnham

**Affiliations:** a Central Alberta Pain and Rehabilitation Institute, Lacombe, Alberta, Canada; b Vivo Cura Health, Calgary, Alberta, Canada; c Department of Clinical Neurosciences, Cumming School of Medicine, University of Calgary, Alberta, Canada; d Division of Physical Medicine and Rehabilitation, University of Alberta, Edmonton, Alberta, Canada

**Keywords:** Platelet rich plasma, Epidural, PRP spine, Regenerative spine, Discogenic low back pain

## Abstract

**Summary of background data:**

Low back pain of disc origin is common yet challenging to treat. Intradiscal platelet rich plasma (PRP) has been advocated, but is associated with risk of discitis. Epidural PRP is less invasive and avoids this risk. Few studies exist evaluating effectiveness and safety of epidural PRP for discogenic low back pain without radiculopathy and the follow-up of the studies tends to be short.

**Objective:**

Prospectively evaluate for 12 months the effectiveness of PRP epidural injections for patients with low back pain without radiculopathy, suspected to be of disc origin.

**Methods:**

11 consecutive patients with refractory low back pain suspected to be of disc origin (compatible clinical assessment; negative lumbosacral medial branch blocks (MBBs) and/or magnetic resonance imaging (MRI) with high intensity zone (HIZ), Modic 1 or 2 changes) participated. Each underwent one (n = 5) or two (n = 6) epidural injections (caudal or interlaminar). The PRP was leukocyte/red cell depleted with an average platelet concentration of ∼2X whole blood. Numerical rating scale (NRS), Pain Disability Quality-Of-Life Questionnaire (PDQQ) score, Oswestry Disability Index (ODI) score, effect on analgesic intake, treatment satisfaction and endorsement were recorded prior to and at 3, 6 and 12-months post-treatment.

**Results:**

Significant improvements in pain and disability were documented post-treatment. Pre-, 3, 6, and 12-month post mean(sd) NRS scores were 7.8(1.8), 5.8(2.7), 5.1(2.5), 4.9(2.8) respectively (F = 7.2; p = 0.002). At 12 months post PRP epidural, the mean improvement in NRS was 36%, 36% had experienced ≥50% pain relief (95% confidence interval (CI): 2%, 70%), and 73% achieved minimal clinically important differences (MCID) (95% CI: 41%, 100%). Similar magnitude improvements in disability (PDQQ and ODI) were documented. At 1-year post, 50% of analgesic users had reduced intake, 91% were satisfied with the treatment and would recommend the procedure to family and friends. No complications were reported.

**Discussions/conclusion:**

This pilot project suggests that PRP epidural injections provide modest yet significant improvements in pain and disability that lasts at least 12 months in patients with low back pain suspected to be of disc origin. Additional research including larger sample size and robust study design is encouraged.

## 1 Introduction

Low back pain of disc origin is common, with estimates that it is the cause of approximately 40% of chronic low back pain and the predominant cause of chronic low back pain in younger patients [1]. However, it remains a challenging condition to treat. No treatment intervention is distinctly superior. Surgical outcomes for disc pain are variable and the role of surgery for discogenic pain is controversial [2,3]. Epidural steroid injections for disc pain without radicular symptoms has not been shown to be effective in most studies and can have significant adverse effects with repeated use [4].

Treatment with biologics such as platelet rich plasma (PRP) may have utility given its anti-inflammatory and regenerative properties [5]. Intradiscal PRP has been advocated as a potential treatment, but does carry the risk of discitis and the potential for adverse effects from needle trauma to the disc. In addition, patients may have multiple symptomatic discs and it can be challenging to know which disc(s) to treat without invasive diagnostic intervention such as provocative discography. Some patients have severe disc space narrowing that makes intradiscal needle entry challenging.

Compared to intradiscal injections, PRP epidurals are less invasive, technically easier, likely carry less risk, and can potentially treat multiple levels as well as adjacent non-disc structures. The majority of published research on PRP epidurals has focused on its potential to treat disc related radiculopathy [[6], [7], [8], [9], [10], [11]]. Early studies suggest favorable results. There is a paucity of research exploring the effect of PRP epidural to treat discogenic low back pain without radiculopathy which is a more common clinical scenario.

The purpose of this study was to explore the effects of epidural platelet rich plasma on patients with refractory low back pain suspected to be of disc origin.

## 2 Methods

This pilot study was carried out in accordance with the code of ethics of the world medical association (Declaration of Helsinki) for experiments involving humans including informed consent and was approved by the Conjoint Health Research Ethics Board of the University of Calgary (REB20-0355). It was conducted at the Central Alberta Pain and Rehabilitation Institute in Lacombe Alberta (CAPRI Clinic). Twelve consecutive patients with chronic refractory low back ± non-radicular leg pain between September 2020 and January 2021 participated. Patients were selected based on a diagnosis of low back pain suspected to be of disc origin. Vertebrogenic pain was included under this umbrella term. The diagnosis of back pain suspected to be of disc origin was made based on a compatible clinical presentation (including no clinical ± MRI evidence of radiculopathy; n = 12), negative lumbar facet medial branch blocks (n = 5) or MRI with Modic type 1 or 2 changes or a high intensity zone/annular fissure (n = 4) or both (n = 2). One patient was lost to follow-up just after the PRP injection. Therefore, the study cohort consisted of 11 patients.

PRP was prepared by drawing 45 mL of venous blood which was centrifuged using a single spin protocol. The blood was centrifuged at 750 G for 5 min. The plasma layer was extracted and used as the PRP. Using this protocol, the PRP is red cell and leukocyte depleted and typically has ∼2x whole blood platelet concentration. The PRP was not activated.

The PRP epidurals were done with image guidance (combined ultrasound and fluoroscopy for the caudal epidurals (n = 9), and fluoroscopy for the interlaminar epidurals(n = 2)). Caudal epidural injections were the preferred route for those without recent MRI scans. For the caudal epidurals, 15–20 mL of PRP was used and for the interlaminar epidurals, 10–15 mL of PRP was used. Six patients underwent a single epidural whereas 6 had a second epidural, most commonly 3–6 months after the first. All epidural injections were performed under strict aseptic precautions. Patient vitals (Blood pressure, pulse rate, oxygen saturation) were taken before and after the procedure. Prior to the epidural injection, local anesthetic (1% lidocaine) was infiltrated into the skin and needle track, but not into the epidural space. Post procedure, patients were advised to avoid unnecessary lifting, bending and exercise for 3 days.

Outcomes were measured using numerical rating scale of pain intensity (NRS), Pain Disability Quality-Of-Life Questionnaire Spine (PDQQ-S), Oswestry Disability Index (ODI) score, effect of intervention on analgesic intake, treatment satisfaction and procedure endorsement. These were measured prior to and at 3-, 6- and 12-months post treatment.

Data analysis included descriptive statistics and repeated measures Analysis of Variance. Questionnaire data were missing for one patient at three months post PRP and one patient at 12 months post PRP. The missing data was dealt with using a last observation carried forward technique.

## 3 Results

Table 1 summarizes the patient characteristics.

**Table 1:** Patient Demographic Data.

Age in years: mean(sd)	48.0 (16.5)
Sex: M:F	3:8
BMI: mean standard deviation (sd)	31.1 (6.2)
Symptom duration in years: mean(sd)	18.4 (14.8)
Pain location: low back; low back and leg	7:4
Regular exercise: yes:no	6:5
Smoker: yes:no	3:8

Following the PRP epidural, there were statistically significant improvements in NRS, PDQQ-S and ODI questionnaire scores reflecting significant improvements in pain, disability and quality-of-life. For the NRS and ODI questionnaires, pre-PRP injection scores were not significantly different (p > 0.05) than the 3-month scores, whereas the 6 and 12-month post PRP injection scores were significantly improved (p < 0.05). The mean improvement in pain at 12 months post PRP injection was 36%. For the PDQQ-S questionnaire, the 6-month post-PRP score was significantly improved compared the pre-PRP score. (Table 2). At 12-months post-PRP, 4 of 11(36%) of patients had ≥50 % pain relief (95% CI: 2%, 70%) and 8 of 11(73%) had achieved minimal clinically important difference (MCID) on NRS (95% CI: 41%, 100%).

**Table 2:** Outcomes.

Outcome measure	Pre PRP Mean(sd)	3month post PRP mean(sd) [%improved]	6month post PRP mean(sd) [%improved]	12month post PRP mean(sd) [%improved]	F	P
**NRS pain**	7.8(1.8)	5.8(2.7) [23.4%]	5.1(2.5) [30.0%]	4.9(2.8) [36.0%]	7.2	0.002
**PDQQ-S**	50.1(6.9)	35.4(18.5) [28.7%]	34.3(16.0) [30.6%]	34.5(16.9) [30.2%]	6.2	0.002
**ODI**	48.2(12.9)	36.0(17.2) [25.1%]	33.0(17.5) [31.1%]	32.0(16.5) [32.2%]	8.3	0.001

At one year post PRP epidural, 3 of 6 patients (50%) who had been taking analgesic medication for their back pain had reduced their analgesic intake. Ten of 11 patients (91%) were satisfied with the treatment and would recommend the procedure to family and friends. There were no reported complications.

## 4 Discussion

The purpose of this prospective case series pilot study was to explore the effect of epidural PRP on patients with refractory low back pain suspected to be of disc origin. We found that, following epidural PRP, modest yet clinically and statistically significant improvements in pain, disability and quality-of-life resulted and were sustained through 6 and 12 months. There was also a trend towards improvement at 3 months post treatment and it's likely that, with a larger sample size, statistical significance would result then as well. Of those patients who were requiring analgesic medication prior to PRP epidural, half were able to reduce intake. The procedure was associated with a high level of patient satisfaction and endorsement. Our results are consistent with previous studies using epidural PRP for low back pain showing significant enduring improvement without adverse events [[12], [13], [14]].

Our study is one of the few to evaluate epidural PRP as a treatment for suspected discogenic low back pain without radiculopathy and to follow outcomes for 12 months. A recent systematic review identified 13 studies between 2016 and 2023 evaluating the clinical application of PRP for epidural therapy [15]. All but one study included patients with radicular pain and the follow-up was 3–6 months in 9 of the 13 studies and one year or longer in only 4 of the studies. The magnitude of the improvements in pain and function are similar to improvements described following intradiscal PRP [15]. Future studies should include a head-to-head comparison of the effectiveness and safety of intradiscal versus epidural PRP to treat discogenic low back pain with extended follow-up. Also, the role of epidural PRP in the treatment of vertebrogenic pain is worth exploring.

We encountered no complications from the procedure. Similarly, studies done to date, as well as the use of epidural blood patches in anesthesia, suggest this is a safe procedure with a low risk of complications Interestingly, one randomized controlled trial documented adverse events following transforaminal injection of corticosteroid for lumbar radiculopathy to be 5 times more common than with growth factors derived from PRP [16]. Although epidural steroid injections are effective in some patients for low back pain, the relief is typically short-lived and the adverse effects of cortisone on bone density are of concern. PRP epidurals appear to have durable therapeutic effects and would not be expected to have any negative effect on bone density.

The ideal PRP formulation for epidural use is unknown. We used a larger volume/lower platelet concentration rather than a smaller/more platelet concentrated volume. We suspected there may be some advantages to using a larger volume including wider spread to adjacent levels and structures., However it may be that a smaller volume (or similar volume) of more concentrated PRP injected at the level of the painful disc will prove more effective. Lastly, the type of epidural approach may affect outcome. Our decision to take a caudal approach for most patients was affected by the favorable results reported by Ruiz-Lopez and Tsai [13], not having recent lumbar MRIs on all patients, and the thought that the procedure could be less costly and more assessable for patients if done by ultrasound. A transforaminal approach has been proven to be more effective than interlaminar or caudal approaches when delivering corticosteroid for disc related radiculopathy and the same may hold true for PRP delivery [17].

This pilot study has significant limitations. The sample size was small which increases the risk of chance findings. There was no control group. Accordingly, the benefits documented following PRP epidural injection cannot be definitively attributed to the treatment itself, as other potentially confounding factors were not controlled for.

Of note is that the mean patient BMI was 31 (median 30) which is higher than the Canadian population mean of 29 [18]. We do not have enough data to say if that is a factor in outcomes. It is possible that some patient factors may predict more favorable outcomes with this procedure (i.e. non obese, nonsmoker, more active).

## 5 Conclusion

This small uncontrolled pilot study suggests that, following PRP epidural injection(s), patients suffering with refractory chronic low back pain suspected to be of disc origin experience modest yet statistically and clinically significant improvements in pain, disability and quality-of-life. These benefits last at least 12 months. Patients are generally satisfied with and endorse the treatment. It may be that PRP epidural is an accessible, relatively low cost and minimally invasive treatment for patients with discogenic low back pain. These results are promising and future research, including controlled studies with larger sample size, is encouraged.

## Declaration of competing interest

None.

## References

[1] Wang F., Cheung C.W., Wong S.S.C. (2023). Regenerative medicine for the treatment of chronic low back pain: a narrative review. J Int Med Res.

[2] Xu W., Ran B., Luo W., Li Z., Gu R. (2021). Is lumbar fusion necessary for chronic low back pain associated with degenerative disk disease? A meta-analysis. World Neurosurg.

[3] Koenders N., Rushton A., Verra M.L., Willems P.C., Hoogeboom T.J., Staal J.B. (2019). Pain and disability after first-time spinal fusion for lumbar degenerative disorders: a systematic review and meta-analysis. Eur Spine J.

[4] Nagpal A., Vu T., Gill B., Conger A., McCormick Z., Duszynski B., Boies B. (2022). Systematic review of the effectiveness of caudal epidural steroid injections in the treatment of chronic low back or radicular pain. Interventional Pain Medicine.

[5] Anitua E., Andia I., Ardanza B., Nurden P., Nurden A.T. (2004). Autologous platelets as a source of proteins for healing and tissue regeneration. Thromb Haemostasis.

[6] Centeno C., Markle J., Dodson E. (2017). The use of lumbar epidural injection of platelet lysate for treatment of radicular pain. J Exp Orthop.

[7] Rawson B. (2020). Platelet-rich plasma and epidural platelet lysate: novel treatment for lumbar disk herniation. J Am Osteopath Assoc.

[8] Demirci A.Y. (2022). The retrospective analysis of platelet-rich plasma and corticosteroid injection under epiduroscopic guidance for radiculopathy in operated or unoperated patients for lumbar disc herniation. Turk J Phys Med Rehabil.

[9] Le V.T., Nguyen Dao L.T., Nguyen A.M. (2023). Transforaminal injection of autologous platelet-rich plasma for lumbar disc herniation: a single-center prospective study in Vietnam. Asian J Surg.

[10] Bise S., Dallaudiere B., Pesquer L. (2020). Comparison of interlaminar CT-guided epidural platelet-rich plasma versus steroid injection in patients with lumbar radicular pain. Eur Radiol.

[11] Wongjarupong A., Pairuchvej S., Laohapornsvan P. (2023). "Platelet-Rich Plasma" epidural injection an emerging strategy in lumbar disc herniation: a Randomized Controlled Trial. BMC Muscoskel Disord.

[12] Bhatia R., Chopra G. (2016). Efficacy of platelet rich plasma via lumbar epidural route in chronic prolapsed intervertebral disc patients-A pilot study. J Clin Diagn Res.

[13] Ruiz-Lopez R., Tsai Y.C. (2020). A randomized double-blind controlled pilot study comparing leucocyte-rich platelet-rich plasma and corticosteroid in caudal epidural injection for complex chronic degenerative spinal pain. Pain Pract.

[14] Correa J., Cortés H., Abella P. (2019). Epidural plasma rich in growth factors for degenerative disc disease: a valuable alternative to conventional “palliative medicine”. Int J Anesth and Clin Med..

[15] Kawabata S., Akeda K., Yamada J. (2023). Advances in platelet-rich plasma treatment for spinal diseases: a systematic review. Int J Mol Sci.

[16] Benítez N.P.P., Hernández C.J.L., García O.T. (2021). Efficacy of parasargital translaminar epidural application of growth factors derived from Platelet Rich Plasma as a treatment for unilateral root pain caused by multisegmental disc disease. Invest Medicoquir.

[17] Kwak S.G., Choo Y.J., Kwak S., Chang M.C. (2023). Effectiveness of transforaminal, interlaminar, and caudal epidural injections in lumbosacral disc herniation: a systematic review and network meta-analysis. Pain Physician.

[18] https://www150.statcan.gc.ca/t1/tbl1/en/tv.action?pid=1310009620&pickMembers%5B0%5D=1.1&pickMembers%5B1%5D=3.1&cubeTimeFrame.startYear=2021&cubeTimeFrame.endYear=2022&referencePeriods=20210101%2C20220101 (last accessed Jan 30, 2024).

